# Naphthalen-1-yl­methanol

**DOI:** 10.1107/S2414314620016466

**Published:** 2020-12-22

**Authors:** Alexander Y. Nazarenko

**Affiliations:** a Chemistry Department, State University of New York, College at Buffalo, 1300 Elmwood Ave, Buffalo, NY 14222-1095, USA; University of Aberdeen, Scotland

**Keywords:** crystal structure, 1-naphthalene­methanol, hydrogen bond

## Abstract

Apart from the OH group, the mol­ecule of the title compound, C_11_H_10_O, is almost planar with all carbon atoms located within 0.03 Å of their mean plane. In the crystal, the mol­ecules are linked by O—H⋯O hydrogen bonds, generating infinite chains running parallel to the [100] direction.

## Abstract 2

The mol­ecules of naphthalen-1-yl­methanol are linked by O—H⋯O hydrogen bonds, generating infinite chains propagating parallel to the [100] direction.

## Graphical abstract

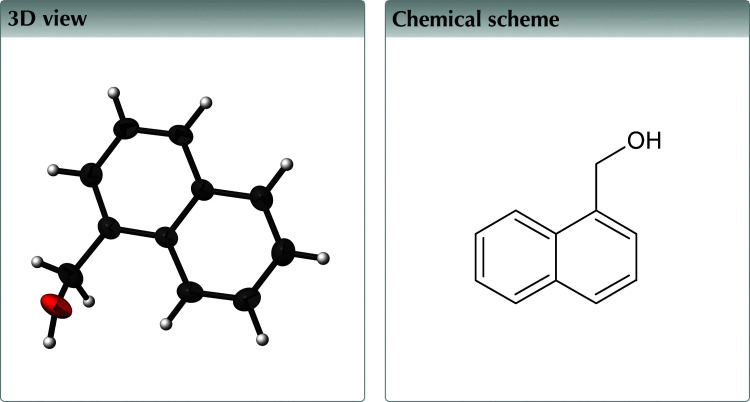

## Structure description

The title compound, C_11_H_10_O, was first prepared by reduction of the corresponding naphthyl­amide (West, 1920 ▸) and by Grignard reaction involving formaldehyde (Ziegler, 1921 ▸). It is available commercially.

**Figure 1:**
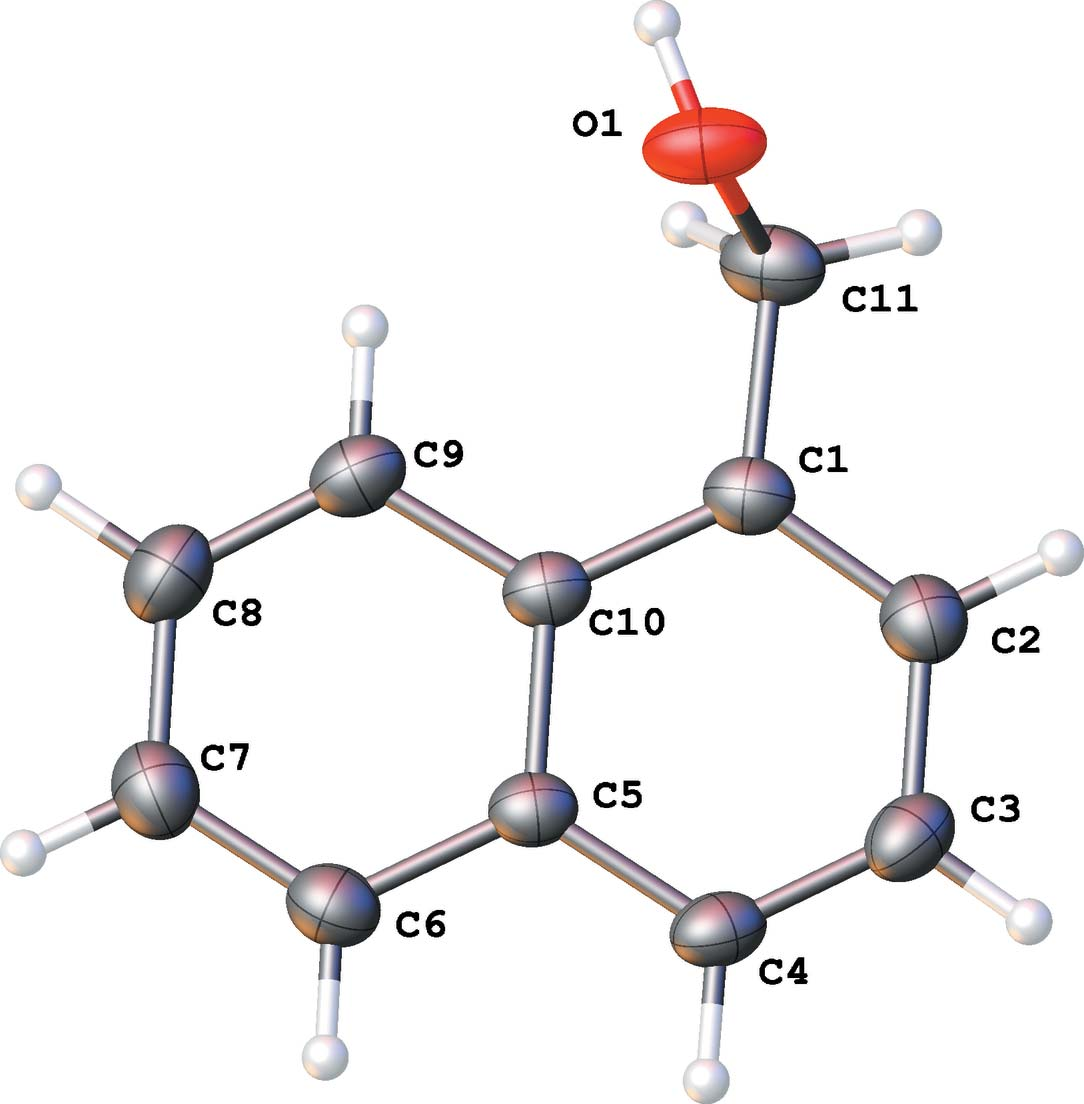
The mol­ecular structure of the title compound with 50% displacement elipsoids.

The title compound (Fig. 1 ▸) exhibits standard bond lengths and angles. Apart from the OH group, the mol­ecule is almost planar: all carbon atoms are located within 0.03 Å of their mean plane and all aromatic hydrogen atoms are also within 0.04 Å of the same plane. Atom O1 is displaced from the mean plane of the other non-hydrogen atoms (r.m.s. deviation = 0.029 Å) by −1.260 (1) Å.

**Figure 2:**
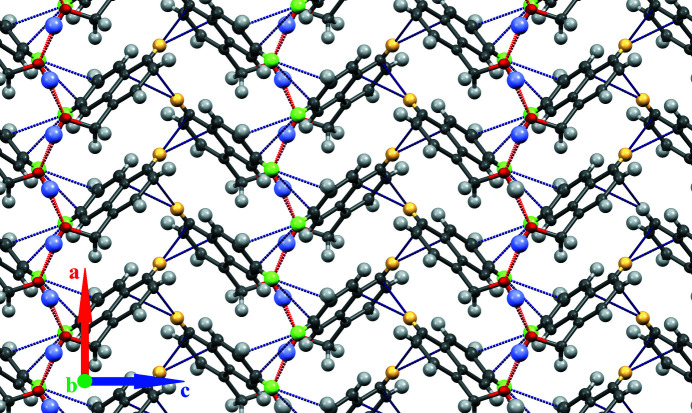
Packing of 1-naphthalene­methanol mol­ecules viewed along the [010] vector. Hydrogen bonds are red, contacts shorter than sum of van der Waals radii are blue. Highlighted hydrogen atoms: H1 (pale blue), H4 (yellow), H8 (green).

In the crystal, the 1-naphthalene­methanol mol­ecules are linked by O1—H1⋯O1^i^ hydrogen bonds (Table 1 ▸, Fig. 2 ▸), generating infinite *C*(2) chains propagating parallel to the [100] direction: adjacent mol­ecules in a chain are related by *a*-glide symmetry. Similar chains were observed in 1-naphthalene­ethanol (Garozzo & Naza­renko, 2016 ▸). Additional C—H⋯C(ar) contacts involving the H4 hydrogen atom and C5 and C4 carbon atoms of another chain help to assemble the chains into a weakly bound layer lying parallel to the (010) plane (Fig. 2 ▸). These layers are held together by van der Waals forces, forming a mol­ecular crystal.

**Figure 3:**
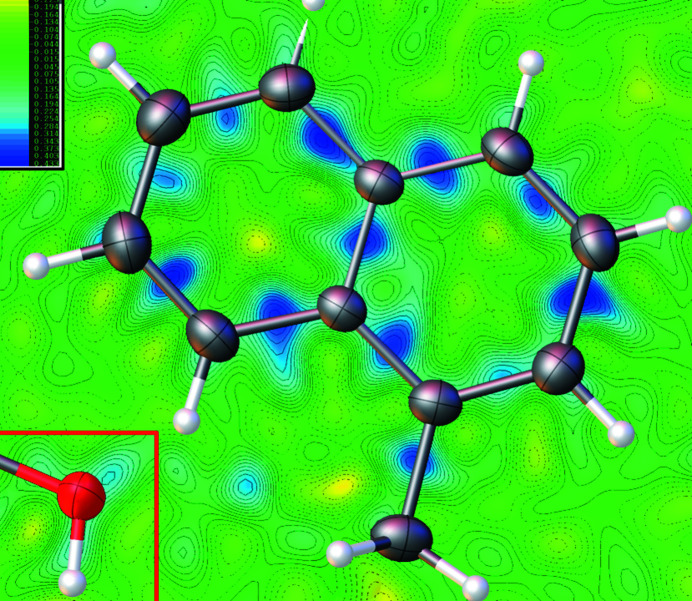
Difference map in the plane of the naphthalene ring system (left lower corner: lone pair area of hydroxyl group).

Difference electron density maps (Fig. 3 ▸) show visible positive density at all covalent bonds and at the lone pair area of the oxygen atom. This effect comes from the limitations of the independent atom model; it results, among other shortcomings, in inflated *R* values and uncertainties of bonding parameters. Application of the Hirshfeld atom refinement with *HARt* (Fugel *et al.*, 2018 ▸) to the same dataset yields a lower *R*(*F*) of 0.036 and significantly lower uncertainties for the bond lengths and angles.

## Synthesis and crystallization

The title compound is commercially available from Aldrich. Recrystallization from ethanol solution yields needle-like crystals, which were used in the current study.

## Refinement

Crystal data, data collection and structure refinement details are summarized in Table 2 ▸

## Supplementary Material

Crystal structure: contains datablock(s) I. DOI: 10.1107/S2414314620016466/hb4373sup1.cif

Structure factors: contains datablock(s) I. DOI: 10.1107/S2414314620016466/hb4373Isup2.hkl

Supporting information file. DOI: 10.1107/S2414314620016466/hb4373Isup3.cdx

Supporting information file. DOI: 10.1107/S2414314620016466/hb4373Isup4.cml

CCDC reference: 2051428

Additional supporting information:  crystallographic information; 3D view; checkCIF report

## Figures and Tables

**Table 1:** Hydrogen-bond geometry (Å, °)

*D*—H⋯*A*	*D*—H	H⋯*A*	*D*⋯*A*	*D*—H⋯*A*
O1—H1⋯O1^i^	0.90 (2)	1.87 (2)	2.7504 (8)	166 (2)

Symmetry code: (i) 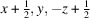 .

**Table 2:** Experimental details

Crystal data	
Chemical formula	C_11_H_10_O
*M*_r_	158.19
Crystal system, space group	Orthorhombic, *P* *b* *c* *a*
Temperature (K)	173
*a*, *b*, *c* (Å)	4.9306 (1), 15.7882 (5), 21.0651 (6)
*V* (Å^3^)	1639.82 (8)
*Z*	8
Radiation type	Mo *K*α
μ (mm^−1^)	0.08
Crystal size (mm)	0.58 × 0.12 × 0.1

Data collection	
Diffractometer	Bruker PHOTON-100 CMOS
Absorption correction	Multi-scan (*SADABS*; Krause *et al.*, 2015 ▸)
*T*_min_, *T*_max_	0.797, 0.862
No. of measured, independent and observed [*I* > 2σ(*I*)] reflections	38547, 2860, 2146
*R*_int_	0.045
(sin θ/λ)_max_ (Å^−1^)	0.747

Refinement	
*R*[*F* ^2^ > 2σ(*F* ^2^)], *wR*(*F* ^2^), *S*	0.051, 0.144, 1.04
No. of reflections	2860
No. of parameters	149
H-atom treatment	All H-atom parameters refined
Δρ_max_, Δρ_min_ (e Å^−3^)	0.35, −0.14

Computer programs: *APEX2* and *SAINT* (Bruker, 2016 ▸), *SHELXT* (Sheldrick, 2015*a* ▸), *SHELXL* (Sheldrick, 2015*b* ▸), *OLEX2* (Dolomanov *et al.*, 2009 ▸) and *Mercury* (Macrae *et al.*, 2020 ▸).

## full crystallographic data

### Crystal data

**Table:**

C_11_H_10_O	*D*_x_ = 1.282 Mg m^−^^3^
*M**_r_* = 158.19	Melting point: 333 K
Orthorhombic, *P**b**c**a*	Mo *K*α radiation, λ = 0.71073 Å
*a* = 4.9306 (1) Å	Cell parameters from 9897 reflections
*b* = 15.7882 (5) Å	θ = 3.2–32.0°
*c* = 21.0651 (6) Å	µ = 0.08 mm^−^^1^
*V* = 1639.82 (8) Å^3^	*T* = 173 K
*Z* = 8	Needle, colourless
*F*(000) = 672	0.58 × 0.12 × 0.1 mm

### Data collection

**Table:**

Bruker PHOTON-100 CMOS diffractometer	2860 independent reflections
Radiation source: sealedtube	2146 reflections with *I* > 2σ(*I*)
Graphite monochromator	*R*_int_ = 0.045
Detector resolution: 10.8 pixels mm^-1^	θ_max_ = 32.1°, θ_min_ = 3.2°
φ and ω scans	*h* = −7→7
Absorption correction: multi-scan (SADABS; Krause *et al.*, 2015)	*k* = −23→23
*T*_min_ = 0.797, *T*_max_ = 0.862	*l* = −31→31
38547 measured reflections	

### Refinement

**Table:**

Refinement on *F*^2^	Primary atom site location: structure-invariant direct methods
Least-squares matrix: full	Hydrogen site location: difference Fourier map
*R*[*F*^2^ > 2σ(*F*^2^)] = 0.051	All H-atom parameters refined
*wR*(*F*^2^) = 0.144	*w* = 1/[σ^2^(*F*_o_^2^) + (0.0706*P*)^2^ + 0.4678*P*] where *P* = (*F*_o_^2^ + 2*F*_c_^2^)/3
*S* = 1.03	(Δ/σ)_max_ < 0.001
2860 reflections	Δρ_max_ = 0.35 e Å^−^^3^
149 parameters	Δρ_min_ = −0.14 e Å^−^^3^
0 restraints	

### Special details

**Table:**

Geometry. All esds (except the esd in the dihedral angle between two l.s. planes) are estimated using the full covariance matrix. The cell esds are taken into account individually in the estimation of esds in distances, angles and torsion angles; correlations between esds in cell parameters are only used when they are defined by crystal symmetry. An approximate (isotropic) treatment of cell esds is used for estimating esds involving l.s. planes.

### Fractional atomic coordinates and isotropic or equivalent isotropic displacement parameters (Å^2^)

**Table:**

	*x*	*y*	*z*	*U*_iso_*/*U*_eq_	
O1	0.64002 (18)	0.45528 (6)	0.27894 (4)	0.0398 (2)	
H1	0.788 (5)	0.4551 (11)	0.2541 (10)	0.068 (5)*	
C1	0.5128 (2)	0.39728 (7)	0.38094 (5)	0.0288 (2)	
C2	0.3861 (2)	0.43905 (7)	0.42978 (5)	0.0330 (2)	
H2	0.441 (3)	0.4976 (9)	0.4386 (7)	0.041 (4)*	
C3	0.1852 (3)	0.39961 (8)	0.46739 (6)	0.0352 (3)	
H3	0.102 (3)	0.4281 (10)	0.5013 (8)	0.045 (4)*	
C4	0.1114 (2)	0.31785 (7)	0.45523 (5)	0.0325 (2)	
H4	−0.027 (3)	0.2898 (9)	0.4812 (7)	0.042 (4)*	
C5	0.2393 (2)	0.27146 (7)	0.40600 (5)	0.0276 (2)	
C6	0.1723 (3)	0.18527 (7)	0.39480 (6)	0.0344 (3)	
H6	0.027 (3)	0.1588 (9)	0.4205 (7)	0.044 (4)*	
C7	0.3045 (3)	0.13985 (8)	0.34904 (6)	0.0388 (3)	
H7	0.259 (4)	0.0829 (11)	0.3412 (8)	0.050 (4)*	
C8	0.5083 (3)	0.17819 (8)	0.31230 (6)	0.0390 (3)	
H8	0.607 (3)	0.1454 (10)	0.2784 (8)	0.050 (4)*	
C9	0.5750 (2)	0.26132 (8)	0.32101 (5)	0.0336 (3)	
H9	0.714 (3)	0.2873 (10)	0.2953 (7)	0.046 (4)*	
C10	0.4438 (2)	0.31084 (7)	0.36840 (5)	0.0268 (2)	
C11	0.7284 (2)	0.44171 (8)	0.34298 (6)	0.0349 (3)	
H11A	0.777 (3)	0.4958 (10)	0.3634 (7)	0.041 (4)*	
H11B	0.902 (3)	0.4052 (9)	0.3427 (7)	0.039 (4)*	

### Atomic displacement parameters (Å^2^)

**Table:**

	*U*^11^	*U*^22^	*U*^33^	*U*^12^	*U*^13^	*U*^23^
O1	0.0254 (4)	0.0620 (6)	0.0319 (4)	−0.0028 (4)	−0.0007 (3)	0.0145 (4)
C1	0.0253 (5)	0.0341 (5)	0.0268 (5)	−0.0015 (4)	−0.0036 (4)	0.0049 (4)
C2	0.0366 (6)	0.0322 (5)	0.0302 (5)	0.0010 (4)	−0.0043 (4)	0.0021 (4)
C3	0.0399 (6)	0.0387 (6)	0.0270 (5)	0.0087 (5)	0.0027 (4)	0.0012 (4)
C4	0.0319 (5)	0.0382 (6)	0.0274 (5)	0.0040 (4)	0.0043 (4)	0.0070 (4)
C5	0.0258 (5)	0.0323 (5)	0.0248 (5)	0.0015 (4)	−0.0012 (4)	0.0056 (4)
C6	0.0354 (6)	0.0333 (5)	0.0343 (6)	−0.0024 (4)	−0.0009 (4)	0.0059 (4)
C7	0.0451 (7)	0.0328 (6)	0.0384 (6)	−0.0006 (5)	−0.0041 (5)	−0.0004 (5)
C8	0.0408 (6)	0.0422 (6)	0.0339 (6)	0.0070 (5)	0.0014 (5)	−0.0049 (5)
C9	0.0285 (5)	0.0433 (6)	0.0290 (5)	0.0017 (4)	0.0020 (4)	0.0011 (4)
C10	0.0231 (4)	0.0330 (5)	0.0242 (4)	0.0014 (4)	−0.0023 (3)	0.0039 (4)
C11	0.0285 (5)	0.0441 (6)	0.0320 (5)	−0.0072 (5)	−0.0046 (4)	0.0072 (5)

### Geometric parameters (Å, º)

**Table:**

O1—H1	0.90 (2)	C5—C10	1.4252 (14)
O1—C11	1.4338 (14)	C6—H6	0.989 (17)
C1—C2	1.3724 (16)	C6—C7	1.3669 (18)
C1—C10	1.4310 (15)	C7—H7	0.940 (17)
C1—C11	1.5039 (16)	C7—C8	1.4054 (19)
C2—H2	0.980 (15)	C8—H8	1.006 (17)
C2—C3	1.4131 (17)	C8—C9	1.3653 (18)
C3—H3	0.939 (16)	C9—H9	0.965 (16)
C3—C4	1.3654 (17)	C9—C10	1.4232 (16)
C4—H4	0.981 (15)	C11—H11A	0.986 (16)
C4—C5	1.4174 (15)	C11—H11B	1.032 (16)
C5—C6	1.4201 (16)		
			
C11—O1—H1	107.5 (14)	C6—C7—H7	120.8 (11)
C2—C1—C10	119.25 (10)	C6—C7—C8	120.22 (11)
C2—C1—C11	119.75 (10)	C8—C7—H7	119.0 (11)
C10—C1—C11	120.97 (10)	C7—C8—H8	121.0 (9)
C1—C2—H2	118.0 (9)	C9—C8—C7	120.80 (11)
C1—C2—C3	121.85 (11)	C9—C8—H8	118.2 (9)
C3—C2—H2	120.1 (9)	C8—C9—H9	120.3 (9)
C2—C3—H3	121.5 (10)	C8—C9—C10	120.87 (11)
C4—C3—C2	119.88 (11)	C10—C9—H9	118.8 (10)
C4—C3—H3	118.6 (10)	C5—C10—C1	118.78 (10)
C3—C4—H4	120.5 (9)	C9—C10—C1	123.06 (10)
C3—C4—C5	120.48 (11)	C9—C10—C5	118.14 (10)
C5—C4—H4	119.0 (9)	O1—C11—C1	110.79 (9)
C4—C5—C6	120.88 (10)	O1—C11—H11A	110.7 (9)
C4—C5—C10	119.74 (10)	O1—C11—H11B	109.3 (8)
C6—C5—C10	119.36 (10)	C1—C11—H11A	110.1 (9)
C5—C6—H6	118.8 (9)	C1—C11—H11B	109.2 (9)
C7—C6—C5	120.60 (11)	H11A—C11—H11B	106.5 (13)
C7—C6—H6	120.6 (9)		
			
C1—C2—C3—C4	−0.39 (18)	C6—C5—C10—C9	−0.45 (15)
C2—C1—C10—C5	1.81 (15)	C6—C7—C8—C9	−0.9 (2)
C2—C1—C10—C9	−176.55 (10)	C7—C8—C9—C10	1.34 (19)
C2—C1—C11—O1	−113.15 (12)	C8—C9—C10—C1	177.73 (11)
C2—C3—C4—C5	1.44 (17)	C8—C9—C10—C5	−0.64 (17)
C3—C4—C5—C6	177.24 (11)	C10—C1—C2—C3	−1.25 (17)
C3—C4—C5—C10	−0.83 (16)	C10—C1—C11—O1	69.09 (14)
C4—C5—C6—C7	−177.20 (11)	C10—C5—C6—C7	0.88 (17)
C4—C5—C10—C1	−0.80 (15)	C11—C1—C2—C3	−179.05 (10)
C4—C5—C10—C9	177.65 (10)	C11—C1—C10—C5	179.58 (9)
C5—C6—C7—C8	−0.22 (19)	C11—C1—C10—C9	1.22 (16)
C6—C5—C10—C1	−178.90 (10)		

### Hydrogen-bond geometry (Å, º)

**Table:**

*D*—H···*A*	*D*—H	H···*A*	*D*···*A*	*D*—H···*A*
O1—H1···O1^i^	0.90 (2)	1.87 (2)	2.7504 (8)	166 (2)

Symmetry code: (i) *x*+1/2, *y*, −*z*+1/2.
